# The complete mitochondrial genome of the tropical oyster *Saccostrea echinata* (Bivalvia: Ostreidae) from the South China Sea

**DOI:** 10.1080/23802359.2020.1869610

**Published:** 2021-02-08

**Authors:** Yafang Li, Lei Xu, Xuehui Wang, Delian Huang, Lianggen Wang, Jiajia Ning, Shuangshuang Liu, Feiyan Du

**Affiliations:** a South China Sea Fisheries Research Institute, Chinese Academy of Fishery Sciences, Guangdong Provincial Key Laboratory of Fishery Ecology and Environment, Guangzhou, China; b Ministry of Agriculture, Key Laboratory of South China Sea Fishery Resources Development and Utilization, Guangzhou, China

**Keywords:** Mitochondrial genome, *Saccostrea echinata*, the South China Sea

## Abstract

*Saccostrea echinata* is a rock perched oyster with wide distribution and tremendous potential for commercial mariculture. However, the taxonomy of this genus is confused. In this study, we described the complete mitochondrial genome of medium-sized form of *Saccostrea echinata.* The genome is 16,282 bp in length, encoding the standard set of 12 protein-coding genes (PCGs), 23 tRNA genes, and two rRNA genes, with circular organization. The overall base composition of the whole mitochondrial genome was A (26.78%), T (36.64%), G (21.99%), and C (14.59%) with an AT bias of 63.42%. The longest PCG of these species was *ND5*, whereas the shortest was *ND4L*.

The giant or blacklip oyster *Saccostrea echinata* (Quoy and Gaimard 1835) that live on rocky shores are broadly distributed throughout the western and south Pacific. It has the commercial potential of mariculture within the Asia-Pacific region due to the suitability of this species for hatchery culture (Glude 1984; Southgate and Peter 1998; Angell et al. 2009). Species in genus Saccostrea are well known for the highly morphological plasticity with different habitat conditions that morphological diagnosis are extremely difficult and time-consuming (Lam and Morton 2006; Amaral and Luiz 2016). DNA-sequenced based methods provide an opportunity to construct additional taxon diagnosis systems that employ DNA sequences as ‘barcodes’ to identify oyster and to conduct further phylogenetic studies (Sekino and Yamashita 2016; Volatiana et al. 2016; Cui 2018; Samantha et al. 2019). Here, we sequenced and annotated mitogenome of *S. echinata* which will provide essential molecular information for the genetical understanding of this oyster and thus contribute to better management and sustainable use of the species.

The specimens of *S. echinata* are collected from Dajin island, northwest of South China Sea (21°52′N, 113°2′E) in 16 September 2020. Whole genomic DNA was extracted from muscle tissue of one specimen of *S. echinata* using TIANamp Marine Animals DNA Kit (TIANGEN, Beijing, China). The concentration for use as a PCR template was adjusted to an *A*_260_ of about 0.05–0.2. The collected specimen and extracted DNA were stored in Guangdong Provincial Key Laboratory of Fishery Ecology and Environment (specimen accession number: JMDJD2020-C3).

DNA sequencing was carried out using genomic DNA by ABI 3730xl DNA automatic sequencer with PCR primers designed from highly conserved regions of transfer RNA (tRNA) sequences of related species (Wu et al. 2019) with additional specific primers designed as required from sequences already obtained. The COI sequence of *S. echinata* was used as reference seeds for iterative assembly by MITObim v.1.8 (Hahn et al. 2013). SeqMan v.7.1.0 was used for the mitogenome assembly and annotation (Swindell and Plasterer 1997). Transfer RNA genes were predicted using online software tRNAScan-SE 1.21 (Lowe and Eddy 1997). All protein-coding gene (PCG) are aligned independently, then concatenated to be applied for phylogenetic reconstruction with other Bivalvia species in MrBayes v 3.12 (Ronquist and Huelsenbeck 2003) using relaxed clock model.

*S. echinata* (MW122840) mitochondrial genome forms a 16,282 bp closed loop. The overall base composition of the whole mitochondrial genome was A (26.78%), T (36.64%), G (21.99%), and C (14.59%) with an AT bias of 63.42%. This mitochondrial genome represents a typical Ostreidae mitochondrial genome and matches with the *Saccostrea kegaki* (KT936590) genome, in which it comprises 12 protein coding gene, 23 tRNA genes, and two rRNA genes (12S rRNA and 16S rRNA) and one A + T-rich region which could also be termed as control region. The 16S rRNA gene is split into two parts by a large fragment. The ATG and GTG initiation codons are used in most of PCGs except *ND41* and *CYTB* (ATA), and the stop codons of all the 12 PCGs were complete. Six PCGs (*COX1*, *CYTB*, *COX2*, *ND1*, *ND4*, and *ND4L*) use TAA as the termination codon; six PCGs (*ND3*, *ND5*, *ND6*, *COX3*, *ATP6*, and *ND2*) use TAG as the termination codon. Meanwhile, the longest PCG of these species was *ND5* (1671 bp), whereas the shortest *ND4L* (281 bp). All the 21 typical tRNAs possess a complete clover leaf secondary structure, ranging from 61 bp to 71 bp. In order to understand the evolutionary relationship and position of *S. echinata*, a Bayesian inference phylogenetic tree was constructed based on mitochondrial PCGs and rRNAs of 21 other Bivalvia species, of which 14 species belong to the same family (Ostreidae) as *S. echinata* and five belong to genus *Saccostrea*. The Bayesian inference phylogenetic tree showed that *S. echinata* (MW122840) firstly grouped with *S. glomerata* (KU310918), *S. kegaki* (KT936590), and *S. mytiloides* (KU310920), then closely related to *S. cucullate* (KP967577) (Figure 1). Support values of Bayesian Posterior were showed on the branches. Phylogenetic analyses consistent with the classification results depended on the morphological characters. We have the confidence to construct phylogenetic trees, based on the complete mitochondrial genomes, but the evolution history of Ostreidae still needs future research to be clearly resolved.

**Figure 1.**
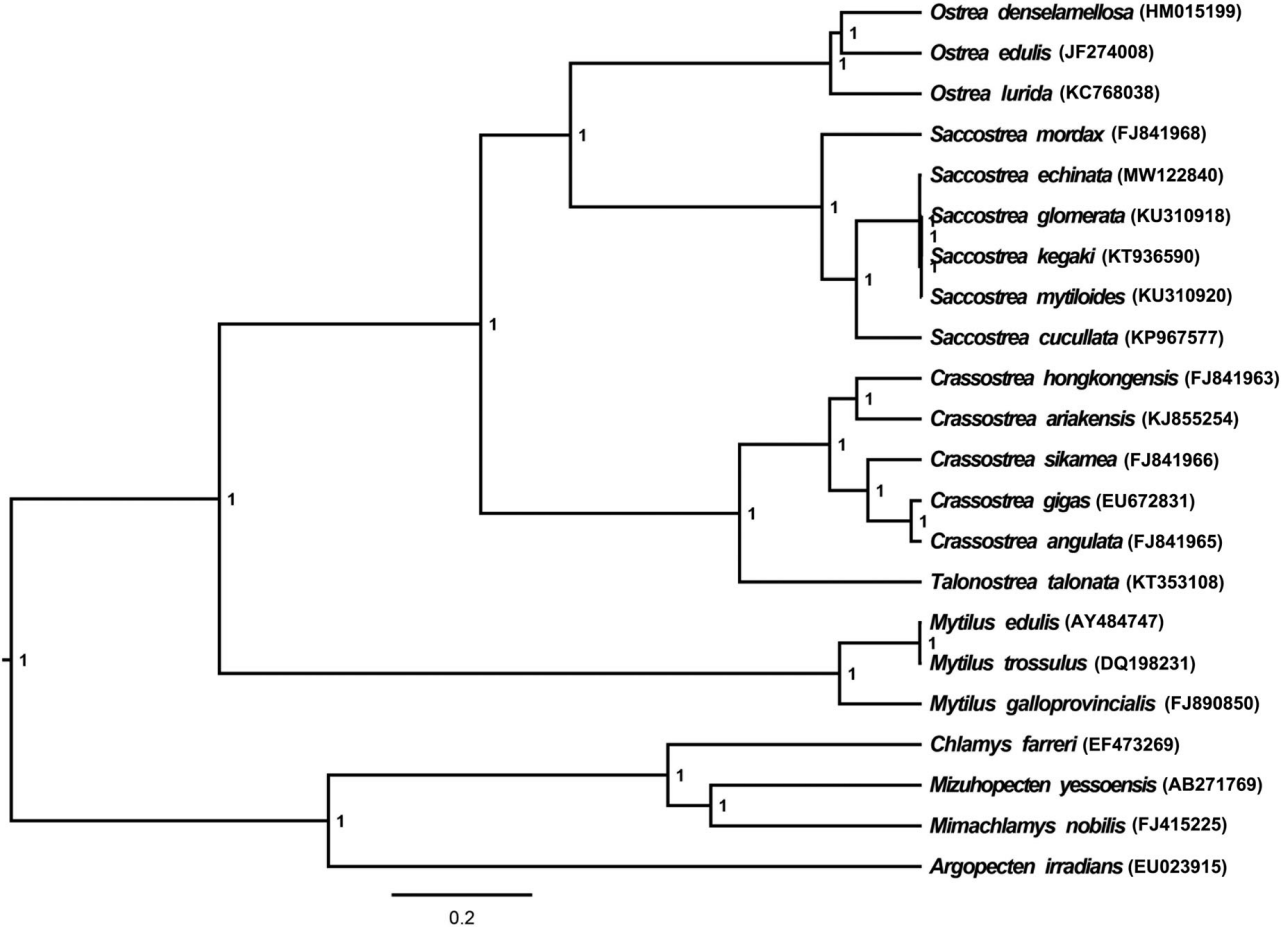
The Bayesian inference phylogenetic tree for 21 Bivalvia species based on mitochondrial PCGs and rRNAs concatenated dataset.

## Data Availability

The genome sequence data that support the findings of this study are openly available in GenBank of NCBI at https://www.ncbi.nlm.nih.gov reference number MW122840. The authors confirm that the data supporting the findings of this study are available at the following link: https://doi.org/10.5281/zenodo.4271298.
